# Suicide, or What?

**Published:** 1859-10

**Authors:** 


### SUICIDE, OR WHAT?

A STATEMENT appears in one of the papers that a clergyman, whose church "had grown with amazing rapidity," was attacked with *inflammation of the lungs.* In spite of the remonstrances of his physician, he stated to his people that he would "continue to preach to them, although he knew his life would be the forfeit," and, doing so, died in a few days after, his physician doing all that medical skill could do to save his life, while the man himself persisted in doing what he had been assured professionally would destroy it. We cannot designate such conduct more appropriately than by saying that it was the fanaticism of a fool, and yet such an one is spoken of as "one of our noblest and truest ministers," and that "a more devoted laborer had not fallen for many years." Although in feeble health, "very much indisposed," he kept working "day and night, notwithstanding the inclement weather, regardless of the remonstrances of his friends. And notwithstanding every attention by the ablest physician, he expired of pneumonia."

If men, who thus unnecessarily, and with such foolhardiness, throw their lives away, let not the intention sanctify the act. And, above all, let not the public press make it out a martyrdom, a self-sacrifice. The most that should be done would be to let it pass in suggestive silence. The cause of religion is not solidly served in any such way. The Almighty is not propitiated by human blood, any more than by Pharisaical boastings, or Hindoo devoteeism. Life, human probation, is an infinite privilege, a talent of priceless value—it is the opportunity of a blissful immortality; and the man who cuts it short, does it at his soul's peril, whether it be done in the slow progress of years, by intemperate, unwise eating or drinking; or whether by its reckless risk in a temporary excitement.

There is such a thing as counting a man's life double. It is done with great propriety at this time in reference to clergymen, when thousands of *organized churches* in our land are in process of dismemberment and extinction for want of men to serve them. Hundreds of such churches are there in New

England alone. To peril a minister's life, under such circumstances, is a double suicide, when there is no adequate necessity for such an exposure.

